# Brain activity changes in a macaque model of oxaliplatin-induced neuropathic cold hypersensitivity

**DOI:** 10.1038/s41598-017-04677-7

**Published:** 2017-06-27

**Authors:** Kazuaki Nagasaka, Kazunori Yamanaka, Shinya Ogawa, Hiroyuki Takamatsu, Noriyuki Higo

**Affiliations:** 1 Human Informatics Research Institute, National Institute of Advanced Industrial Science and Technology (AIST), Tsukuba, Ibaraki 305-8568 Japan; 2 Graduate School of Comprehensive Human Sciences, University of Tsukuba, Tsukuba, Ibaraki 305-8577 Japan; 3 Pharmacology Group, Hamamatsu Pharma Research, Inc., Hamamatsu, Shizuoka 431-2103 Japan

## Abstract

The antineoplastic agent oxaliplatin induces a painful peripheral neuropathy characterized by an acute cold hypersensitivity. There is a lack of effective treatments to manage oxaliplatin-induced cold hypersensitivity which is due, in part, to a lack of understanding of the pathophysiology of oxaliplatin-induced cold hypersensitivity. Thus, brain activity in oxaliplatin-treated macaques was examined using functional magnetic resonance imaging (fMRI). Oxaliplatin treatment reduced tail withdrawal latency to a cold (10 °C) stimulus, indicating cold hypersensitivity and increased activation in the secondary somatosensory cortex (SII) and the anterior insular cortex (Ins) was observed. By contrast, no activation was observed in these areas following cold stimulation in untreated macaques. Systemic treatment with an antinociceptive dose of the serotonergic-noradrenergic reuptake inhibitor duloxetine decreased SII and Ins activity. Pharmacological inactivation of SII and Ins activity by microinjection of the GABA_A_ receptor agonist muscimol increased tail withdrawal latency. The current findings indicate that SII/Ins activity is a potential mediator of oxaliplatin-induced cold hypersensitivity.

## Introduction

Chemotherapy-induced peripheral neuropathy (CIPN) is a common adverse side effect in patients receiving anticancer drugs, and greatly diminishes quality of life^1, 2^. Currently, there are no analgesics approved specifically for use in CIPN^3^. Oxaliplatin, an anticancer chemotherapeutic used for the treatment of advanced colorectal cancer, has a distinctive neurotoxicity that is observed following each infusion—an acute peripheral neuropathy characterized by cold hypersensitivity^4–6^. The severity of the cold hypersensitivity is such that it could lead to early discontinuation of potentially beneficial chemotherapy. Therefore, therapeutics are needed to manage oxaliplatin-associated CIPN.

A number of drugs, including pregabalin, extensively used in the management of various neuropathic pains, have demonstrated significant antinociceptive efficacy in rodent models of oxaliplatin-induced peripheral neuropathy^7^. However, to date, clinical trials have not supported preclinical findings^3^. One source for the lack of confidence in the preclinical models could be the phylogenetic distance between rodents and humans. In an attempt to improve translation of preclinical findings to clinically useful treatments, a preclinical model of oxaliplatin-induced peripheral neuropathy was recently developed in macaques, which exhibited a robust, dose-dependent, early-onset and short-lasting cold hypersensitivity^7^, paralleling clinical findings^1, 8^. Interestingly, the effect of clinical analgesics on cold hypersensitivity in the macaque model parallel clinical findings; treatment with the serotonergic-norepinephrine reuptake inhibitor duloxetine ameliorated oxaliplatin-induced cold hypersensitivity, as it did in a phase III clinical study^9^ whereas the pregabalin did not^7, 10^.

While behavioral endpoints are crucial, the relationship between pain-related behaviors and *in vivo* brain activation related to pain observed in nonhuman animals has not been fully elaborated. Combining brain activity with behavioral endpoints could lead to better understanding of the role of the brain in pain processing than focusing on either process alone. Moreover, evaluating the extent of involvement of the cerebral cortex is important in bridging the gap between preclinical pain assessment in nonhuman species and clinical pain assessment in human patients, considering that the cerebral cortex is crucial in humans for conscious somatosensory perception^11^. Additionally, while pain itself is a subjective experience, brain activity could be useful as a quantifiable marker of pain to define efficacy and elaborate drug mechanism^12^.

Our hypothesis is that oxaliplatin leads to cold pain (or cold hypersensitivity) that is mediated through brain activation and that deactivation (or inhibition) decreases oxaliplatin-induced cold pain. Thus, the primary objective of the current study is to identify regional brain activity in macaques with oxaliplatin-induced peripheral neuropathy using functional magnetic resonance imaging (fMRI). The macaque has an advantage over other experimental animal species in that brain areas involved in pain processing are similar to that of humans^13–17^. This homology between macaques and humans, in combination with the relatively large brain in macaques, allows for direct comparison of imaging data between macaques and humans. The present study also investigated modulation of brain activity in oxaliplatin-treated macaques by systemic administration of an antinociceptive dose of duloxetine and by microinjection of the GABA_A_ receptor agonist muscimol in order to confirm a potential association between brain activity and behavior.

## Results

### Cold hypersensitivity and brain activity in oxaliplatin-treated macaques

**Figure 1:**
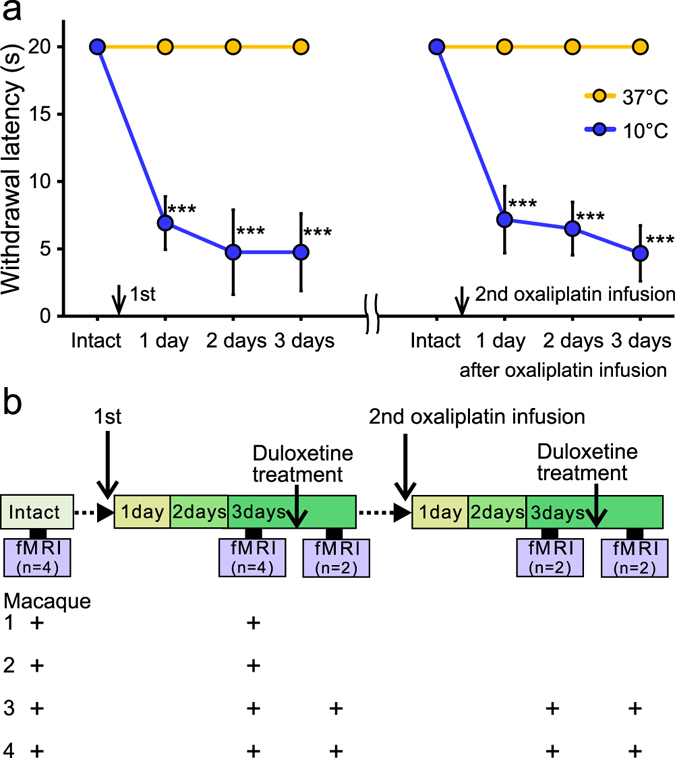
Cold hypersensitivity in oxaliplatin-treated macaques and fMRI study experimental design. (**a**) Effect of oxaliplatin treatment on tail withdrawal latency over time. One treatment cycle is a two week period; there were 14 days between i.v. infusions of oxaliplatin. Withdrawal latencies to a 10 °C stimulus in either the first or second cycle were significantly decreased compared with pre-infusion withdrawal latency (“Intact”). By contrast, oxaliplatin treatment did not significantly change withdrawal latency to 37 °C water. Values are expressed as mean ± SD. ***P < 0.001, compared with “Intact” (one-way repeated measures analysis of variance followed by Bonferoni’s post-hoc test). (**b**) Brain activity was assessed before (“intact”, n = 4) and 3 days after oxaliplatin infusion (n = 4), using a Philips Ingenia 3.0 T MRI system. Functional magnetic resonance imaging scans were also performed 1 hour after oral administration of duloxetine at 3 days after either the first or second infusion of oxaliplatin (n = 2). Plus signs indicate the experimental treatment of each macaque used in fMRI experiments. Macaques were sedated by continuous intravenous infusion of propofol during scanning.

Cold hypersensitivity was observed after each infusion of oxaliplatin in the macaques used in the present study (Fig. 1a), as was observed in a previous study^7^. Three days after oxaliplatin infusion, the tail withdrawal latency to cold (10 °C) was significantly decreased compared with pre-infusion withdrawal latency, indicating hypersensitivity to cold. To identify regional brain activity in macaques with oxaliplatin-induced peripheral neuropathy characterized by cold hypersensitivity, fMRI scans were performed (Fig. 1b).

**Figure 2:**
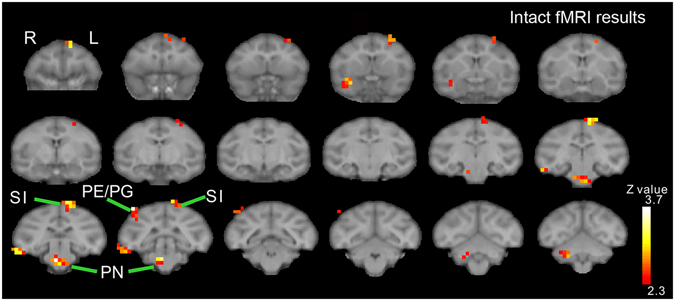
Brain activity in intact macaques. In untreated (“intact”) macaques, increased brain activity associated with a cold (10 °C) stimulus was observed in several brain areas including the parietal (PE/PG) and primary somatosensory cortices (SI) and pontine nuclei (PN).

Before oxaliplatin infusion, brain activity associated with a cold stimulus in macaques was observed using fMRI. In untreated (“intact”) macaques, 10 °C increased activity in several brain areas including the parietal and primary somatosensory cortices and pontine nuclei (Fig. 2, Table 1), which are also activated by skin-cooling in healthy humans^18, 19^. Thus, somatostimulation in the macaque activated homologus human brain structures.

**Table 1:** Brain areas showing significantly increased activation before and after oxaliplatin infusion.

Area	Hemisphere	Z value	x	y	z (mm)
Intact (10 °C–37 °C)					
PE/PG	Right	3.99	−18	−2	16
PN	Right	3.85	−2	0	−12
SI	Left	3.49	4	2	20
PMd	Left	3.44	4	26	16
	Left	2.99	12	18	16
TEd	Right	3.43	−24	0	−6
TG	Right	3.18	−12	18	−8
Cb	Right	3.04	−8	−10	−12
Post-oxaliplatin (10 °C–37 °C) > Intact (10 °C–37 °C)					
SII/Ins	Right	3.64	−16	20	2
	Left	3.10	14	16	2
Cb	Left	3.04	8	−8	−8

Z -value of the peak voxels are indicated. The locations are indicated with the positions along the x (ipsi-contralateral), y (rostro-caudal) and z (dorso-ventral) axes of the Horsley-Clarke stereotaxic coordinates. Cb, cerebellum; PMd, dorsal premortor cortex; TEd, dorsal inferotemporal cortex; Ins, Insular cortex; PE/PG, PE/PG of the inferior parietal cortex; PN, pontine nuclei; SI, primary somatosensory cortex; SII, secondary somatosensory cortex; TG, temporal pole.

**Figure 3:**
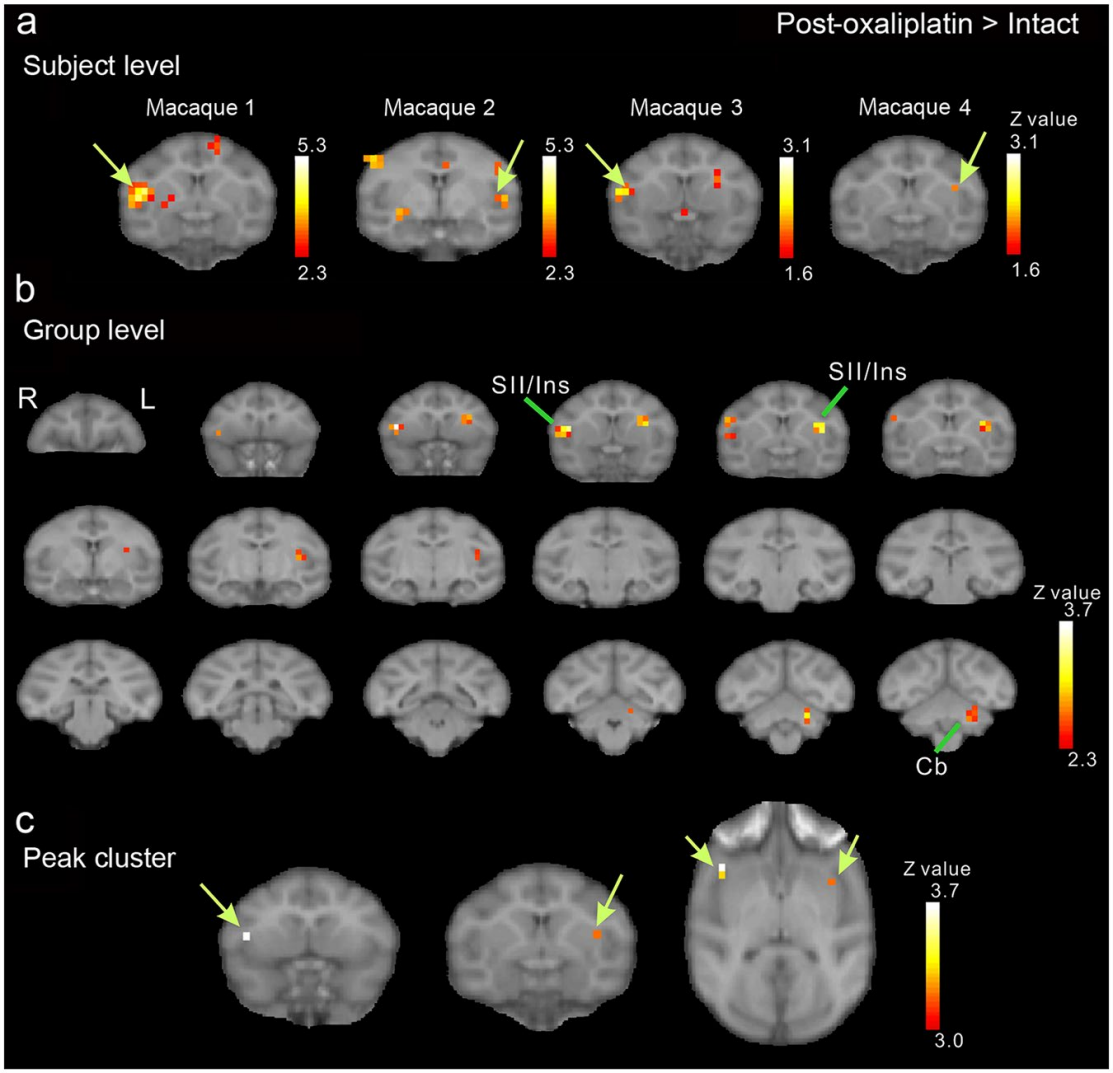
Brain activity changes associated with oxaliplatin-induced cold hypersensitivity. Three days after oxaliplatin infusion, increased activation in the secondary somatosensory cortex (SII) and the insular cortex (Ins) in both hemispheres, compared with activation before oxaliplatin infusion (“Intact”) (c.f. Table 1), were associated with low-temperature stimulation (i.e., activity in response to 10 °C stimulation minus activity in response to 37 °C (control) stimulation). (**a**) Results of subject-level analysis. Increased activation in SII/Ins (arrows) is observed in all four oxaliplatin-treated macaques. (**b**) Results of group-level analysis. A series of coronal slices, spaced 2 mm apart, are arranged from rostral (upper left, y = 24) to caudal (lower right, y = −10). Cb, cerebellum. (**c**) Peak voxels were located in the anterior SII/Ins of both hemispheres (arrows). The arrows indicate peak voxels in each hemisphere.

Three days after oxaliplatin infusion, fMRI scans showed that cold stimulation-related activation was enhanced in several brain areas. Increased activation in the secondary somatosensory cortex (SII) and the insular cortex (Ins) is shown in the analysis of fMRI data from each macaque (Fig. 3a). In the group-level analysis, the clusters with the two most significant changes in brain activation were located bilaterally in SII/Ins (Fig. 3b, Table 1). The peak voxels of the clusters were located in the anterior SII/Ins of both hemispheres (Fig. 3c), and the cluster in the left hemisphere extended into posterior SII/Ins (Fig. 3b). The cluster with the third most significant change in activation was located in the left cerebellum (Fig. 3b, Table 1).

### Changes in brain activity after duloxetine treatment in oxaliplatin-treated macaques

**Figure 4:**
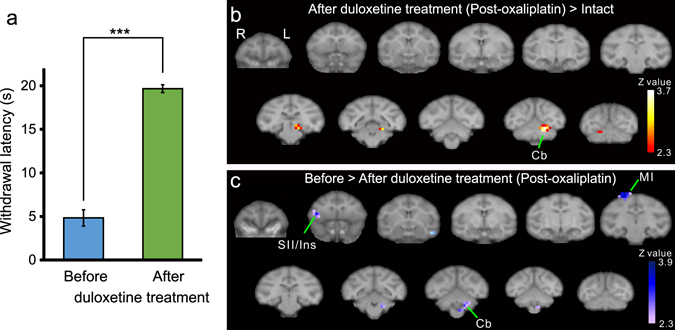
Effect of duloxetine on brain activity in oxaliplatin-treated macaques. (**a**) Systemic duloxetine ameliorated oxaliplatin-induced cold hypersensitivity; withdrawal latencies to 10 °C in either cycle were significantly increased (***P < 0.001, Paired t-test). Values are expressed as mean ± SD. (**b**) After duloxetine treatment, voxels in the secondary somatosensory cortex (SII) and the insular cortex (Ins) were no different compared to that of intact (pre-oxaliplatin infusion) macaques, (P < 0.01, uncorrected). (**c**) Activation in the SII and Ins was significantly decreased after duloxetine treatment, compared with brain activity before duloxetine treatment (P < 0.01, uncorrected; Table 4). In (**b**) and (**c**), a series of coronal slices, spaced by 4 mm, are arranged from rostral (upper-left, y = 24) to caudal (lower-right, y = −16). Cb, cerebellum; MI, primary motor cortex.

Systemic duloxetine treatment ameliorates cold hypersensitivity in oxaliplatin-treated macaques^7^. It is possible that duloxetine treatment leads to changes in brain activity, in parallel with the antinociceptive effect of duloxetine. Thus, fMRI scans were performed following duloxetine administration in oxaliplatin-treated macaques (Fig. 1b). Duloxetine treatment (30 mg/kg, *p*.*o*.; Fig. 4a) significantly increased withdrawal latency, in line with previous findings^7^. Following duloxetine treatment in oxaliplatin-treated macaques, no cold-induced enhancement of SII/Ins activation was observed (Fig. 4b, Table 2), in contrast to the robust SII/Ins activation observed in oxaliplatin-treated macaques that were not treated with duloxetine (Fig. 3, Table 1). In fact, compared with brain activity before duloxetine treatment, significantly decreased activation was found in several brain areas, the SII/Ins in particular, following duloxetine treatment (Fig. 4c, Table 2). These results suggest that the antinociceptive effect of duloxetine in oxaliplatin-induced cold hypersensitivity could be in part through a reduction of elevated activation in SII/Ins.

**Table 2:** Brain areas showing significant changes in activation after duloxetine treatment in oxaliplatin-treated macaques.

Area	Hemisphere	Z value	x	y	z (mm)
After duloxetine treatment (Post-oxaliplatin, 10 °C–37 °C) > Intact (10 °C–37 °C)					
Cb	Left	3.63	8	−12	−6
V1	Left	3.43	−10	24	2
TFO	Left	3.12	8	−2	−6
Before > After duloxetine treatment (Post-oxaliplatin, 10 °C–37 °C)					
STG	Left	3.89	20	20	−10
MI	Right	3.49	−8	4	20
SII/Ins	Right	3.35	−16	22	2
Cb	Left	2.93	6	−8	−12

Z -value of the peak voxels are indicated. The locations are indicated with the positions along the x (ipsi-contralateral), y (rostro-caudal) and z (dorso-ventral) axes of the Horsley-Clarke stereotaxic coordinates. Cb, cerebellum; Ins, Insular cortex; MI, primary motor cortex; SII, secondary somatosensory cortex; STG, superior temporal gyrus; TFO, area TFO of the parahippocampal cortex; V1, primary visual cortex.

### Pharmacological inactivation of brain activity

**Figure 5:**
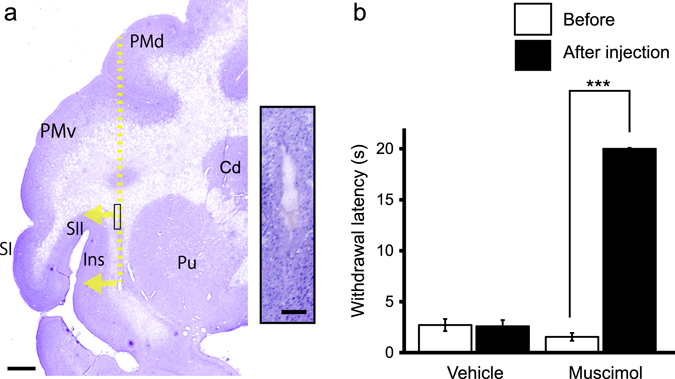
Effect of bilateral inactivation of the secondary somatosensory cortex (SII) and the insular cortex (Ins) on oxaliplatin-induced cold hypersensitivity. (**a**) Representative Nissl-stained section showing brain microinjection sites from oxaliplatin-treated macaques. The yellow dotted line indicates the needle track, and arrows indicate the direction of muscimol diffusion. Scale bar = 2 mm. The inset is a high-magnification photomicrograph of the needle track in the area shown by the box on the low-magnification photomicrograph. The injection sites were confirmed to be located within white matter beneath SII/Ins, within 2 mm from the boundary of the gray matter, in both muscimol-injected macaques and the vehicle-injected macaque. Scale bar in the inset = 200 μm. Cd. Caudate nucleus; PMd, dorsal premortor cortex; PMv, ventral premortor cortex; Pu, putamen; SI, primary somatosensory cortex. (**b**) Bilateral microinjection of muscimol to SII/Ins in oxaliplatin-treated macaques significantly increased mean withdrawal latency to 10 °C cold (***P < 0.001, compared to pre-muscimol injection (“Before”) Paired t-test), indicating an involvement of SII/Ins in cold hypersensitivity. No change in the withdrawal latency was observed in a vehicle-injected oxaliplatin-treated macaque. Values are expressed as mean ± SD.

To investigate a potential involvement of SII/Ins activity with cold hypersensitivity, SII and Ins were transiently inactivated by a focal microinjection of 3.0 µL of the GABA_A_ receptor agonist muscimol. This volume of muscimol has been shown to inactivate a spherical volume of brain tissue roughly 5–6 mm in diameter^20^. In oxaliplatin-treated macaques, muscimol was microinjected into 2 sites centered at the middle of the anterior SII/Ins (Fig. 5a) in each hemisphere. Following muscimol microinjection, withdrawal latencies to cold stimulation were significantly increased compared with pre-microinjection withdrawal latencies (Fig. 5b), suggesting that SII/Ins activation mediates oxaliplatin-induced cold hypersensitivity.

## Discussion

The current study demonstrated robust changes in brain activity, as observed via fMRI, in parallel with changes in pain-related behaviors in the macaque, a species that is phylogenetically close to humans. Oxaliplatin treatment induced a robust hypersensitivity to cold, as indicated by decreased withdrawal latency to cold stimulation, and a concurrent increase in SII/Ins activity. Systemic treatment with an antinociceptive dose of duloxetine reduced SII/Ins activity to levels observed in normal, untreated macaques. Indirect support for SII/Ins involvement in the current model is a marked antinociceptive effect following microinjection of an analog of an inhibitory neurotransmitter, muscimol, into the SII/Ins.

A potential confound of the current study was the use of propofol to anesthetize the macaques during fMRI scans. Propofol was used during fMRI scanning to prevent motion artifacts, which could confound detection of brain activation. A previous fMRI experiment in rats that were sedated with propofol showed that the increases in blood oxygenation level-dependent (BOLD) signal intensity during somatosensory stimulation was partially dampened relative to that observed during the awake state. Nonetheless, a robust signal was still present with propofol anesthesia^21^. In another fMRI study that utilized rats under propofol anesthesia, reliable changes were detected in signal intensity in the somatosensory cortex during median nerve stimulation^22^. Furthermore, propofol sedation has little effect on cortical somatosensory evoked potentials in humans^23, 24^. Together with the fact that the analgesic effect of propofol is extremely weak^25, 26^, propofol sedation is appropriate for detecting pain-related activation patterns in the brain. Indeed, the present fMRI study demonstrated significantly increased brain activity associated with oxaliplatin-induced neuropathic cold hypersensitivity—activation in the SII/Ins—and this activation is consistent with clinical brain imaging studies, wherein activation of SII/Ins is associated with allodynia and cutaneous hypersensitivity in chronic neuropathic pain patients^27–30^.

One other limitation that should be pointed out is that the number of macaques used in the present fMRI study was small compared to those used in most human fMRI studies, and a fixed-effect model was used for group analysis of data from two to four macaques. Such a model was also used in previous macaque fMRI studies wherein the number of macaques was kept low for practical considerations^31–33^. Nonetheless, the current analysis found increased activation in SII/Ins after oxaliplatin infusion within each macaque. Moreover, increased withdrawal latencies in oxaliplatin-treated macaques following microinjection of muscimol into SII/Ins reinforces the notion that SII/Ins activation is in part responsible for cold hypersensitivity.

The activation of SII/Ins in patients with neuropathic pain can occur in the contralateral and/or ipsilateral hemispheres relative to the painful side^28^. Although the present fMRI findings showed that the stimulation-related cluster of activation in the left hemisphere was more caudally extended than that in the right hemisphere, it is not entirely clear why this was so as the stimulus was applied to a midline structure, the tail. A future study that applies somatosensory stimulation to the macaque’s legs or arms could clarify whether laterality or bilaterality is observed in oxaliplatin-induced cold hypersensitivity.

Duloxetine has shown significant efficacy against oxaliplatin-induced neuropathic pain in a phase III clinical trial^3, 9^ yet its analgesic mechanism of action has yet to be determined. The pharmacological mechanism of duloxetine is blocking synaptic reuptake of serotonin and norepinephrine, increasing levels of these neurotransmitters, thereby reducing pain^34^. Whether this occurs directly, at SII/Ins^35–38^ or indirectly through, for example, modulation of brainstem serotonin and norepinephrine neurons that project to cortical areas^39^, remains to be elucidated. A peripheral site of action could also play a role as duloxetine blocks peripheral nerve-expressed sodium channel function^40^.

At the same time, the current nonhuman primate model combined with brain imaging could be used to understand why drugs used to manage other types of neuropathic pain do not show efficacy in oxaliplatin-induced neuropathic pain. Furthermore, perhaps the basis for the differential pharmacological profile between rodent models and the nonhuman primate model could be a fundamental difference in brain activation. Uncovering the relationship between pharmacodynamics and brain anatomy and physiology in each species should lead to greater understanding of the mechanism of drug efficacy across species. It is suggested that data collected from a wide range of species could be used to guide the development of novel therapeutics for the treatment of oxaliplatin-induced neuropathic pain.

To date, there are no approved drugs for the management of CIPN. While a number of pharmacotherapeutics used clinically to manage painful peripheral neuropathies demonstrated efficacy in rodent oxaliplatin-induced models, none, except for duloxetine, have demonstrated clinical efficacy^3, 10^. A potential reason why the development of novel analgesics to treat CIPN has been unsuccessful to date, is because the rodent CIPN models have poor clinical predictivity. In terms of brain anatomy and function, including those of the SII/Ins, the macaque shares greater similarities with humans than rodents^17, 41^. Brain activity, as measured by fMRI, in the macaque, combined with behavioral outcomes could be used to further understand the mechanism of oxaliplatin-induced neuropathic pain. While speculative, the current study suggests that the current nonhuman primate model could contribute to narrowing the translational gap between preclinical and clinical pain research.

## Methods

### Subjects

A total of seven (four for fMRI scan, two for muscimol microinjection, and one for vehicle microinjection) male adult cynomolgus macaques (*Macaca fascicularis*), weighing 2.4–3.8 kg, were used in the present study. No statistical method was used to pre-determine sample sizes. The fewest number of animals was used on the basis of ethical considerations and data similarity; our sample sizes are similar to those reported in previous publications^7, 20, 42^. The macaques were purchased from a local supplier (Shin Nippon Biomedical Laboratories, Ltd., Kagoshima, Japan).

### Ethics statements

All study procedures were reviewed and approved by the Hamamatsu Pharma Research, Inc., Animal Care and Use Committee and the Institutional Animal Care and Use Committee of the National Institute of Advanced Industrial Science and Technology (AIST), and were carried out in accordance with guidelines within the “Guide for the Care and Use of Laboratory animals” (Eighth ed., National Academy of Sciences). The macaques were housed in adjoining individual primate cages that allowed social interactions. Room conditions, including humidity, temperature, and light were monitored daily by research and animal care staff. Animals had free access to tap water and were fed standard nonhuman primate chow (Oriental Yeast Co., Ltd., Chiba, Tokyo, Japan), which was supplemented weekly with fresh fruits or vegetables. The animals’ home cages were supplied with enrichment and positive interaction (e.g. hand feeding of treats) between research and animal care staff.

### Drug administration and fMRI scan

Oxaliplatin (Pfizer, Tokyo, Japan) was dissolved in 5% glucose/water solution (Otsuka Pharmaceutical Co., Ltd., Tokushima, Japan) and intravenously (*i*.*v*., 5 mg/kg) infused over a period of 2 h in accordance with instructions for clinical administration (oxaliplatin, package insert, Pfizer, Tokyo, Japan). Brain activity was assessed in four macaques (Fig. 1a) before and 3 days after oxaliplatin infusion, using a Philips Ingenia 3.0 T MRI system (Philips Ingenia 3.0 T, Philips Healthcare, Best, The Netherlands). The anatomical MRI protocols consisted of a T1-weighted turbo field echo sequence (repetition time (TR)/echo time (TE), 7.3/3.2 ms; number of excitations (NEX), 2; flip angle, 8°; field of view, 134 mm × 134 mm; matrix, 224 × 224; slice thickness, 0.6 mm; number of slices, 200). Functional scan sequences consisted of field-echo, echo-planar imaging (TR/TE, 3000/35 ms; flip angle, 90°; field of view, 120 mm × 120 mm; matrix, 64 × 64; slice thickness, 2.0 mm; number of slices, 27).

The macaques were sedated by continuous intravenous infusion of propofol (0.4 mg/kg/min), which has little, if any, analgesic effect^25, 26^. During each fMRI scan, a gel pack (Hakugen-Earth, Tokyo, Japan) at either 10 °C or 37 °C was applied by hand. The 10 °C or 37 °C stimuli were alternately applied to the tail for 30 sec and a 30 sec interval separated each stimulus presentation. Forty blocks, each consisting of the two conditions, either 10 °C or 37 °C stimulation, were applied. Therefore, the tail was stimulated 40 times at each temperature both before and 3 days after oxaliplatin infusion for each macaque. A digital timer mediated the timing of the placement and removal of the stimuli (Presentation software; Neurobehavioral Systems Inc., San Francisco, CA, USA).

Among the four oxaliplatin-treated macaques used for fMRI scanning, the effect of serotonergic–noradrenergic reuptake inhibitor duloxetine HCl on acute cold hypersensitivity was evaluated in two macaques (Fig. 1a). Duloxetine HCl (Tokyo Chemical Industry Co., Ltd., Tokyo, Japan), was dissolved in distilled water (Otsuka Pharmaceutical Co., Ltd., Tokushima, Japan) on the day of testing. The macaques received 30 mg/kg (in 5 mL/kg, *p*.*o*.) of duloxetine, and MRI scanning was performed 1 h following dosing using the procedure described above. Duloxetine treatment was performed 3 days after either the first or second infusion of oxaliplatin (Fig. 1b), when maximal cold hypersensitivity was observed. No sedation was observed with the currently tested dose of duloxetine.

### Analysis of MRI data

Images were skull-stripped using Brain Extraction Tool (BET) in FSL (FMRIB, Oxford, UK). All subsequent analyses were conducted with SPM12 software (Wellcome Department of Cognitive Neurology, London, UK). The images were realigned and resliced on to the mean EPI image to correct for head motion. The EPI images were co-registered to the corresponding the T1-weighted anatomical image, and normalized to a macaque brain template^43^. The resulting image was smoothed with a 4 mm × 4 mm × 4 mm full-width at half-maximum Gaussian kernel. Voxel-wise statistical analysis was based on a general linear model. A fixed-effect model was used for group analysis of data from the four macaques. The contrast (10 °C stimulation – 37 °C stimulation before oxaliplatin infusion) was defined to isolate regions responsive to 10 °C stimulation-related signals in the intact brain. Contrast was also defined as (10 °C stimulation – 37 °C stimulation after oxaliplatin infusion) – (10 °C stimulation – 37 °C stimulation before oxaliplatin infusion). This contrast examines regions in which 10 °C stimulation-related signals increased after the infusion of oxaliplatin. In addition, contrasts were defined as (10 °C stimulation – 37 °C stimulation after duloxetine infusion in oxaliplatin-infused macaques) – (10 °C stimulation – 37 °C stimulation before oxaliplatin infusion), and (10 °C stimulation – 37 °C stimulation before duloxetine infusion in oxaliplatin-infused macaques) – (10 °C stimulation – 37 °C stimulation after duloxetine infusion in oxaliplatin-infused macaques), in order to examine regions in which 10 °C stimulation-related signals decreased after duloxetine administration. Peaks were considered significant at a voxel threshold of Z > 2.3 (*P* < 0.01, uncorrected for multiple comparisons, one-tailed) or Z > 3.0 (*P* < 0.001, uncorrected for multiple comparisons, one-tailed) and a cluster extent of eight voxels. These analyses of fMRI data in the present study were similar to that in a previous imaging study using positron emission tomography^20^.

### Behavioral scoring

The tail immersion test was performed to demonstrate changes in cutaneous sensitivity following oxaliplatin treatment^7^. Briefly, previously habituated macaques were restrained in a monkey chair. Hair from the distal 10 cm of the tail was removed with a hair clipper. The distal tail was immersed in water at 10 °C or 37 °C (control) in random order, and tail withdrawal latencies were timed with a stop-watch in 1 sec increments, up to a maximum (cut-off) latency of 20 sec. If an animal does not remove its tail from the water by 20 sec, the tail was removed from the water, and the cut-off latency was assigned. Three latencies were obtained, about 1 min apart, and the average latency was reported.

### Microinjection of muscimol

Brain microinjection of muscimol was performed on two macaques, using a procedure similar to that used in a previous report^20^. A craniotomy was made over SII/Ins of both hemispheres under isoflurane anesthesia. Muscimol, a GABA_A_ receptor agonist (5 µg/µL, dissolved in 0.1 M phosphate buffer at pH 7.4; Sigma, St. Louis, MO), was slowly injected with pressure via a microsyringe (MS-10, Ito Corporation, Fuji, Japan). The tip of the injection needle was located just medial to the anterior SII/Ins, and the orifice of the injection needle was oriented laterally, in order to allow injected muscimol to diffuse toward SII/Ins (Fig. 5a). Three µL of muscimol was then injected at 2 sites centered at the middle of the anterior SII/Ins, separated by 5 mm in a dorsoventral direction, for each hemisphere. After injection, the needle was retained for 10 min to allow the solution to disperse and minimize backflow through the needle track. The effect of muscimol on cold hypersensitivity was evaluated using the tail immersion test as described above. Testing was conducted 2 hours after muscimol injection, when the macaques were fully recovered from isoflurane anesthesia. There was no apparent impairment in movement, and the macaques immediately withdrew their tail when the distal tail was immersed in water at 60 °C, as they did before muscimol injection (data not shown). An equivalent volume of vehicle (0.1 M phosphate buffer at pH 7.4) was microinjected into one oxaliplatin-treated macaque, into the same brain areas; no change in withdrawal latency was observed in the vehicle-microinjected macaque.

The location of the needle tracks was confirmed histologically in Nissl-stained brain sections. Tissue preparation was performed as previously described^44–46^. Briefly, the macaques were deeply anesthetized with sodium pentobarbital and then perfused through the ascending aorta with ice-cold saline, followed by ice-cold fixative consisting of 4% paraformaldehyde in phosphate buffer (pH 7.4). After successive incubation in sucrose solutions, the brain was frozen and coronal sections (16 μm thickness) were made at the level of SII/Ins. Images of Nissl-stained sections were acquired with an Olympus BX60 microscope equipped with a 3CCD color video camera and digitized with an image analysis system (StereoInvestigator, MBF Bioscience, Williston VT, USA).

### Statistical analysis

Values are expressed as mean ± SD. Changes over time in the oxaliplatin-treated macaques were analyzed using a one-way repeated measures analysis of variance followed by Bonferoni’s test for post hoc comparisons (Graphpad Prizm 6, Graph Pad Software Inc., San Diego, CA). For statistical analysis, differences were considered significant at P < 0.05.
